# Supplementary material for: Once-Weekly Insulin Icodec in Diabetes Mellitus: A Systematic Review and Meta-Analysis of Randomized Clinical Trials (ONWARDS Clinical Program)
Source: Biomedicines. 2024 Aug 14;12(8):1852. doi: 10.3390/biomedicines12081852 (PMC11352070; doi:10.3390/biomedicines12081852)
Supplement: Supplementary file 1 [file biomedicines-12-01852-s001.zip › biomedicines-3152124-supplementary.pdf]

Once-Weekly Insulin Icodec In Diabetes Mellitus: a Systematic Review And Meta-Analysis of Randomized Clinical Trials (ONWARDS Clinical Program)

Giuseppe Lisco[1], Anna De Tullio[1], Vincenzo De Geronimo[2], Vito Angelo Giagulli[1], Edoardo Guastamacchia[1], Giuseppina Piazzolla[1], Olga Eugenia Disoteo[3]*, Vincenzo Triggiani[1]

[1] Interdisciplinary Department of Medicine, School of Medicine, University of Bari "Aldo Moro", Piazza Giulio Cesare 11, 70124 Bari, BA, Italy

[2] Unit of Endocrinology, Policlinico Morgagni CCD, 95125 Catania, Italy

[3] Unit of Endocrinology, Diabetology, Dietetics and Clinical Nutrition, Sant Anna Hospital, 22020 San Fermo della Battaglia – Como, Italy.

*Corresponding author

ORCid

G.L. 0000-0001-6521-8578

A. DT. 0000-0001-6341-6140

V. DG. 0000-0002-0271-985

E.G. 0000-0001-8666-8658

G.P. 0000-0003-3221-6562

O.E.D. 0000-0002-9369-2629

V.T. 0000-0001-6308-0528

Scopus id

G.L. 57203761410

A. DT. 57215385465

V. DG. 57219991434

E.G. 6701775432

G.P. 55944806100

O.E.D. 55218012200

V.T. 6603380071

Figure S1. PRISMA 2020 flow diagram for the systematic review

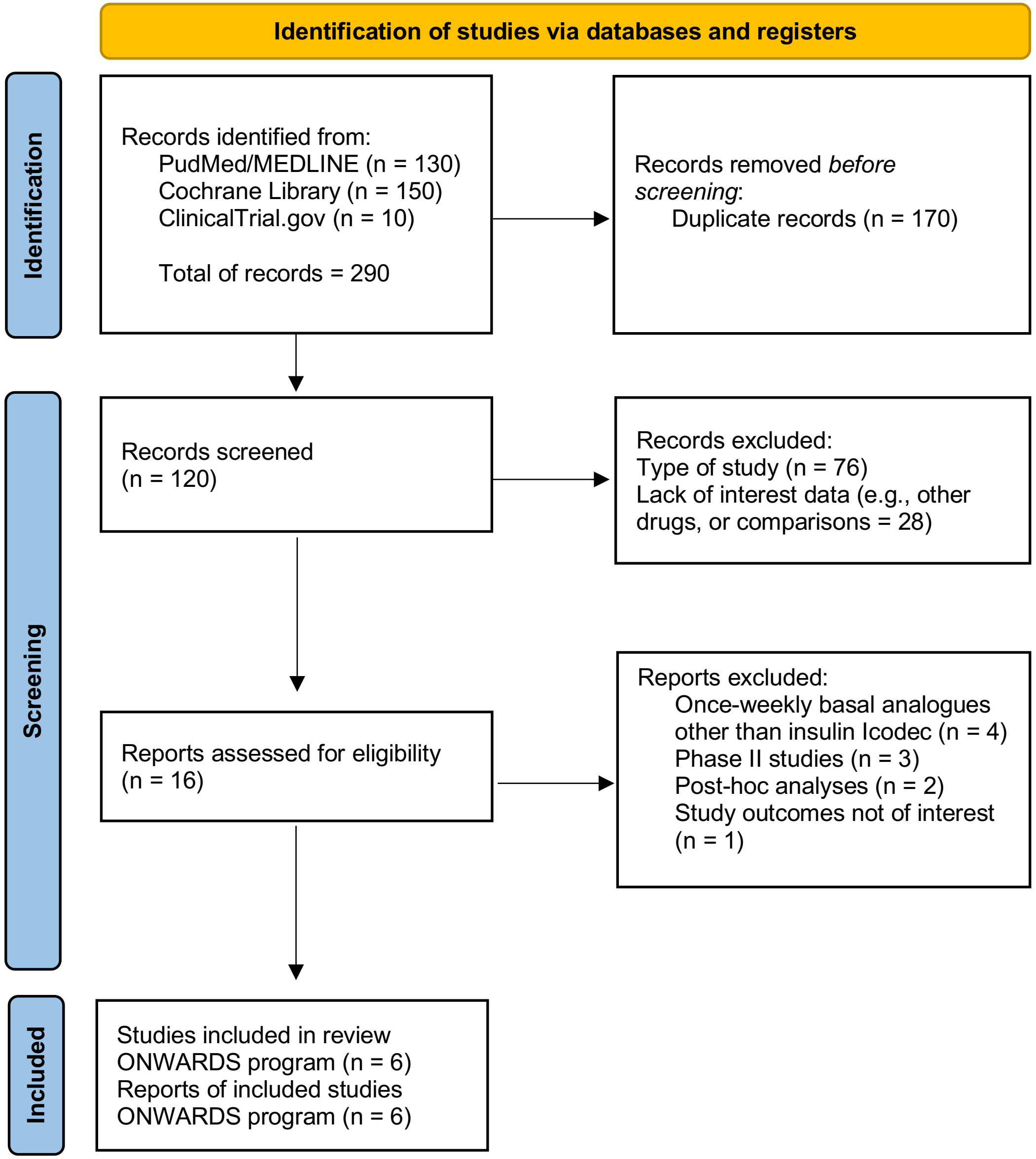

Figure S2. Funnel plot for the estimation of publication bias.

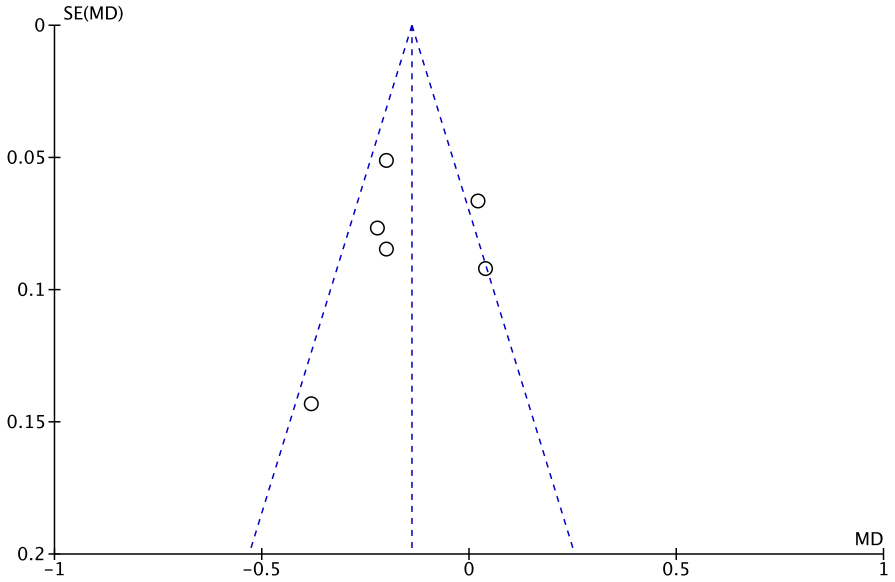

Figure S3. Forest plot of meta-analysis for mean change in fasting plasma glucose levels (ETD, mg/dL) from baseline to study completion (intention-to-treat analysis).

	Icodec			Once−daily basal insulin				Mean Difference
**Study or Subgroup**	**Mean**	**SD**	**Total**	**Mean**	**SD**	**Total**	**Weight**	**IV, Random, 95% CI**
ONWARDS 4	−32	49.9119	291	−29.52	49.9119	291	17.7%	−2.48 [−10.59, 5.63]
ONWARDS 1	−60.32	37.2111	492	−60.08	37.2111	492	24.2%	−0.24 [−4.89, 4.41]
ONWARDS 3	−54	37.1161	294	−54	37.1161	294	21.6%	0.00 [−6.00, 6.00]
ONWARDS 2	−28.47	33.8569	260	−29.18	33.8569	260	21.9%	0.71 [−5.11, 6.53]
ONWARDS 6	−15	61.5433	290	−34	61.5433	292	14.6%	19.00 [9.00, 29.00]
**Total (95% CI)**			**1627**			**1629**	**100.0%**	**2.44 [−2.95, 7.82]**

Heterogeneity: $Tau^2 = 25.57$; $Chi^2 = 13.51$, df = 4 (P = 0.009); $I^2 = 70\%$
Test for overall effect: Z = 0.89 (P = 0.37)

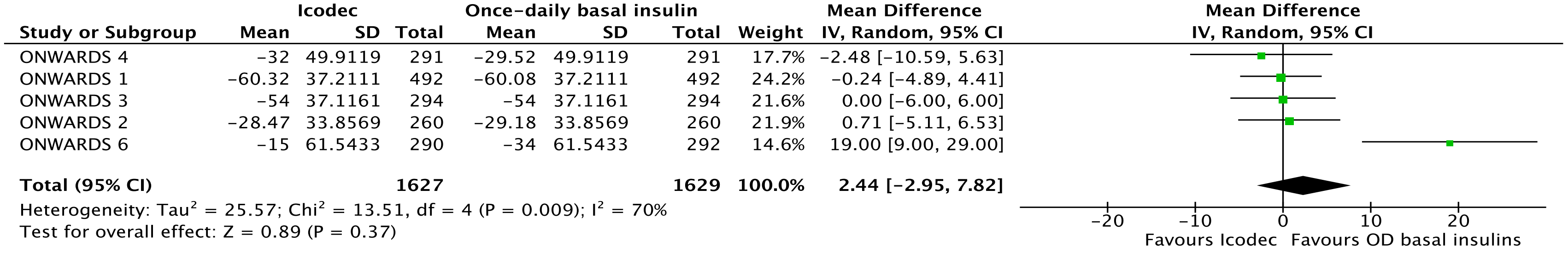

Abbreviations: ETD, Estimated Treatment Difference; OD, Once-Daily.

Figure S4. Forest plot of meta-analysis for mean change in time in range (ETD, %) throughout the last 4 weeks of trials (intention-to-treat analysis).

Study or Subgroup	Icodec Mean	Icodec SD	Icodec Total	Once-daily basal insulin Mean	Once-daily basal insulin SD	Once-daily basal insulin Total	Weight	Mean Difference IV, Random, 95% CI
ONWARDS 6	59.1	12.7791	290	60.8	12.7791	290	28.8%	−1.70 [−3.78, 0.38]
ONWARDS 4	66.9	18.5862	291	66.4	18.5862	291	25.5%	0.50 [−2.52, 3.52]
ONWARDS 2	63.1	24.7379	238	59.5	24.7379	239	20.4%	3.60 [−0.84, 8.04]
ONWARDS 1	71.9	24.6473	492	66.9	24.6473	492	25.3%	5.00 [1.92, 8.08]
**Total (95% CI)**			**1311**			**1312**	**100.0%**	**1.64 [−1.65, 4.93]**

Heterogeneity: $Tau^2 = 8.68$; $Chi^2 = 14.25$, df = 3 (P = 0.003); $I^2 = 79\%$
Test for overall effect: Z = 0.98 (P = 0.33)

Mean Difference IV, Random, 95% CI

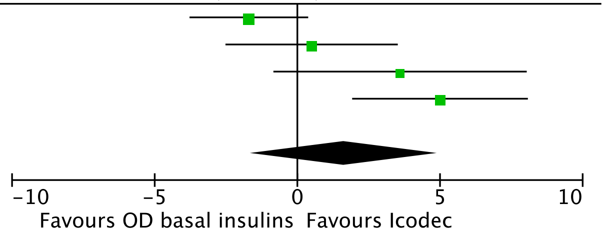

Abbreviations: ETD, Estimated Treatment Difference; OD, Once-Daily.

Figure S5. Forest plot of meta-analysis for mean change in time above range (ETD, %) throughout the last 4 weeks of trials (intention-to-treat analysis).

Study or Subgroup	Icodec Mean	Icodec SD	Icodec Total	Once-daily basal insulin Mean	Once-daily basal insulin SD	Once-daily basal insulin Total	Weight	Mean Difference IV, Random, 95% CI
ONWARDS 1	26.9	19.2857	492	32.3	19.2857	492	28.2%	−5.40 [−7.81, −2.99]
ONWARDS 2	35.5	25.5737	238	39.7	25.5737	239	19.7%	−4.20 [−8.79, 0.39]
ONWARDS 4	30.5	18.9555	291	31.3	18.9555	291	25.5%	−0.80 [−3.88, 2.28]
ONWARDS 6	37	17.2937	290	36.2	17.2937	292	26.6%	0.80 [−2.01, 3.61]
**Total (95% CI)**			**1311**			**1314**	**100.0%**	**−2.34 [−5.51, 0.83]**

Heterogeneity: $Tau^2 = 7.75$; $Chi^2 = 12.56$, df = 3 (P = 0.006); $I^2 = 76\%$
Test for overall effect: Z = 1.45 (P = 0.15)

Mean Difference IV, Random, 95% CI

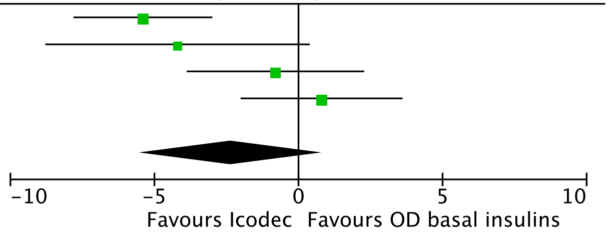

Abbreviations: ETD, Estimated Treatment Difference; OD, Once-Daily.

Figure S6. Forest plot of meta-analysis for probability of experiencing any adverse event (ERR, %) from baseline to study completion.

Study or Subgroup	Icodec Events	Icodec Total	Once-daily basal insulin Events	Once-daily basal insulin Total	Weight	Risk Ratio M-H, Random, 95% CI
ONWARDS 1	397	492	389	492	39.3%	1.02 [0.96, 1.09]
ONWARDS 4	171	291	167	291	8.1%	1.02 [0.89, 1.18]
ONWARDS 6	240	290	236	292	26.3%	1.02 [0.95, 1.11]
ONWARDS 5	279	542	270	538	11.2%	1.03 [0.91, 1.15]
ONWARDS 3	177	293	167	294	8.4%	1.06 [0.93, 1.22]
ONWARDS 2	161	262	134	263	6.7%	1.21 [1.04, 1.40]
**Total (95% CI)**		**2170**		**2170**	**100.0%**	**1.04 [1.00, 1.08]**
Total events	1425		1363			

Heterogeneity: $Tau^2 = 0.00$; $Chi^2 = 4.49$, df = 5 (P = 0.48); $I^2 = 0\%$
Test for overall effect: Z = 1.82 (P = 0.07)

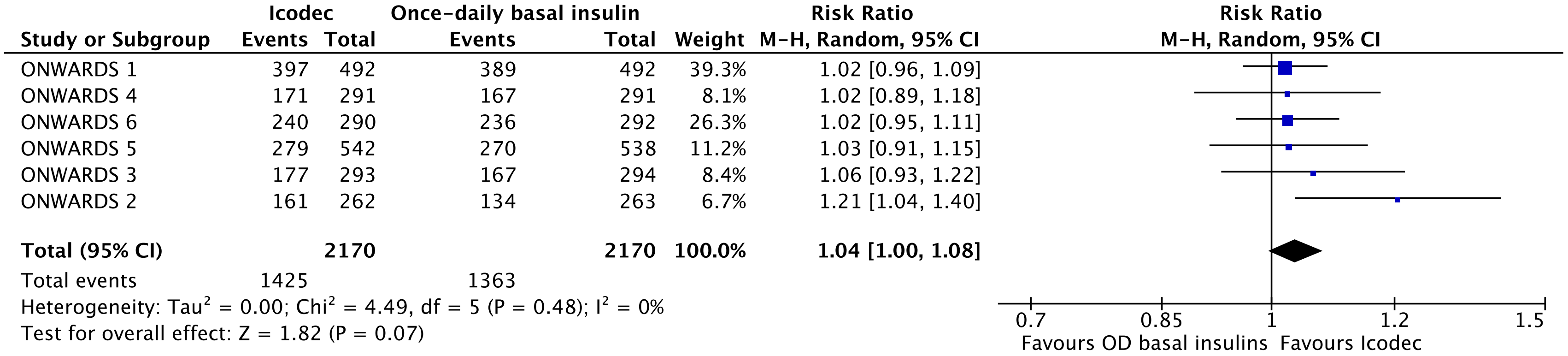

Abbreviations: ERR, Estimated Risk Ratio; OD, Once-Daily.

Figure S7. Forest plot of meta-analysis for probability of experiencing any adverse event (ERR, %) probably or possibly related to insulin use from baseline to study completion.

Study or Subgroup	Icodec Events	Icodec Total	Once-daily basal insulin Events	Once-daily basal insulin Total	Weight	Risk Ratio M-H, Random, 95% CI
ONWARDS 3	27	293	32	294	14.7%	0.85 [0.52, 1.38]
ONWARDS 1	76	492	72	492	30.3%	1.06 [0.78, 1.42]
ONWARDS 5	29	542	24	538	12.8%	1.20 [0.71, 2.03]
ONWARDS 4	32	291	24	291	13.9%	1.33 [0.81, 2.21]
ONWARDS 6	50	290	33	292	19.4%	1.53 [1.01, 2.30]
ONWARDS 2	25	262	13	263	9.0%	1.93 [1.01, 3.69]
**Total (95% CI)**		**2170**		**2170**	**100.0%**	**1.22 [0.99, 1.49]**
Total events	239		198			

Heterogeneity: $Tau^2 = 0.01$; $Chi^2 = 6.26$, df = 5 (P = 0.28); $I^2 = 20\%$
Test for overall effect: Z = 1.87 (P = 0.06)

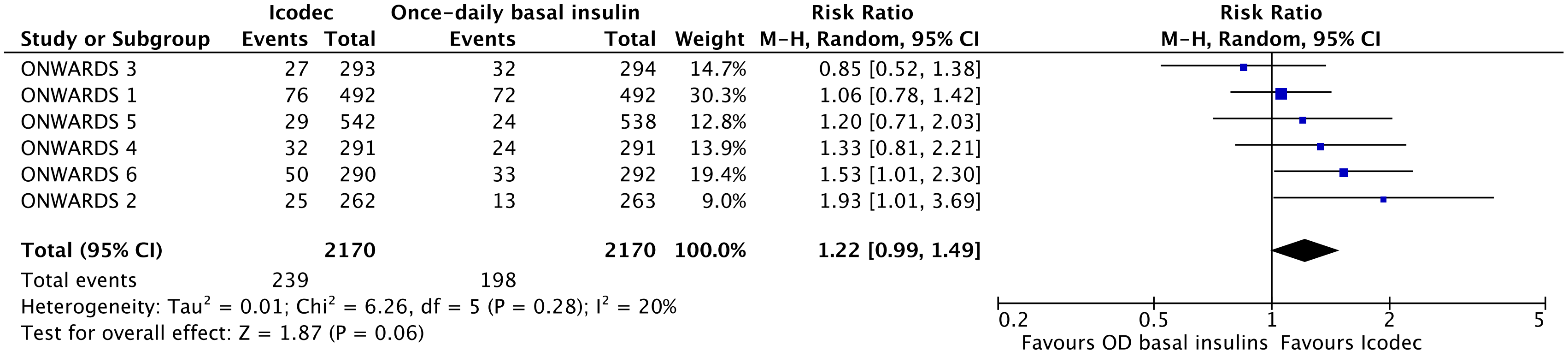

Abbreviations: ERR, Estimated Risk Ratio; OD, Once-Daily.

Figure S8. Forest plot of meta-analysis for probability of experiencing serious adverse event (ERR, %) from baseline to study completion.

Study or Subgroup	Icodec Events	Icodec Total	Once-daily basal insulin Events	Once-daily basal insulin Total	Weight	Risk Ratio M-H, Random, 95% CI
ONWARDS 5	45	542	57	538	25.4%	0.78 [0.54, 1.14]
ONWARDS 4	22	291	25	291	11.7%	0.88 [0.51, 1.52]
ONWARDS 1	64	492	71	492	35.7%	0.90 [0.66, 1.23]
ONWARDS 3	15	293	15	294	7.2%	1.00 [0.50, 2.01]
ONWARDS 6	24	290	20	292	10.8%	1.21 [0.68, 2.14]
ONWARDS 2	22	262	16	263	9.1%	1.38 [0.74, 2.57]
**Total (95% CI)**		**2170**		**2170**	**100.0%**	**0.94 [0.78, 1.13]**
Total events	192		204			

Heterogeneity: $Tau^2 = 0.00$; $Chi^2 = 3.29$, df = 5 (P = 0.66); $I^2 = 0\%$
Test for overall effect: Z = 0.67 (P = 0.50)

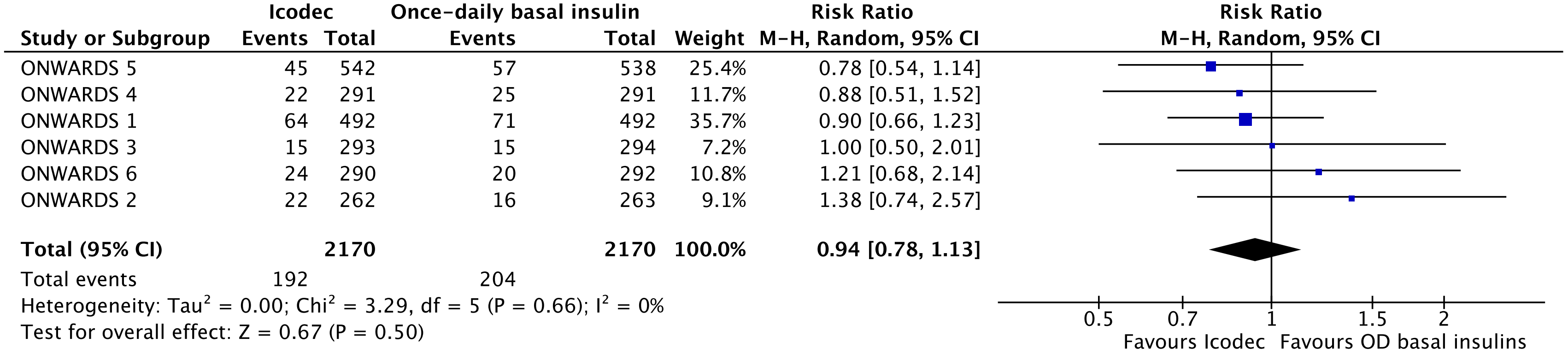

Abbreviations: ERR, Estimated Risk Ratio; OD, Once-Daily.

Figure S9. Forest plot of meta-analysis for probability of experiencing serious adverse event (ERR, %) probably or possibly related to insulin use from baseline to study completion.

Study or Subgroup	Icodec Events	Icodec Total	Once-daily basal insulin Events	Once-daily basal insulin Total	Weight	Risk Ratio M-H, Random, 95% CI
ONWARDS 3	0	293	0	294		Not estimable
ONWARDS 2	0	262	1	263	2.3%	0.33 [0.01, 8.18]
ONWARDS 1	64	492	71	492	70.3%	0.90 [0.66, 1.23]
ONWARDS 5	2	542	2	538	5.8%	0.99 [0.14, 7.02]
ONWARDS 4	2	291	2	291	5.8%	1.00 [0.14, 7.05]
ONWARDS 6	12	290	4	292	15.8%	3.02 [0.99, 9.26]
**Total (95% CI)**		**2170**		**2170**	**100.0%**	**1.08 [0.66, 1.76]**
Total events	80		80			

Heterogeneity: $Tau^2 = 0.06$; $Chi^2 = 4.62$, df = 4 (P = 0.33); $I^2 = 13\%$
Test for overall effect: Z = 0.31 (P = 0.76)

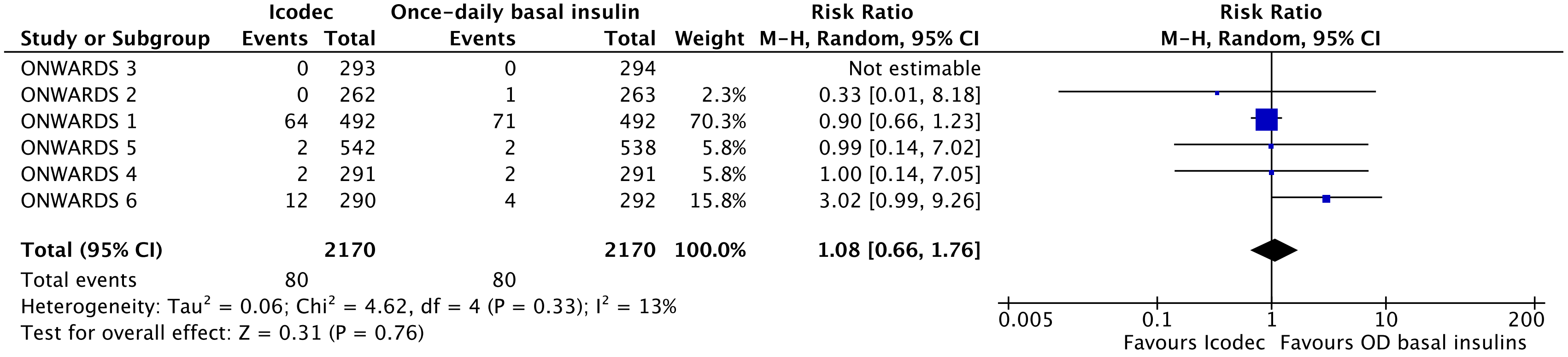

Abbreviations: ERR, Estimated Risk Ratio; OD, Once-Daily.

Figure S10. Forest plot of meta-analysis for probability of experiencing injection-site reaction (ERR, %) from baseline to study completion.

Study or Subgroup	Icodec Events	Icodec Total	Once-daily basal insulin Events	Once-daily basal insulin Total	Weight	Risk Ratio M-H, Random, 95% CI
ONWARDS 6	1	290	2	292	5.1%	0.50 [0.05, 5.52]
ONWARDS 1	7	492	12	492	25.1%	0.58 [0.23, 1.47]
ONWARDS 5	5	542	7	538	18.5%	0.71 [0.23, 2.22]
ONWARDS 4	2	291	2	291	7.5%	1.00 [0.14, 7.05]
ONWARDS 3	25	293	13	294	38.1%	1.93 [1.01, 3.70]
ONWARDS 2	3	262	1	263	5.7%	3.01 [0.32, 28.76]
**Total (95% CI)**		**2170**		**2170**	**100.0%**	**1.08 [0.62, 1.90]**
Total events	43		37			

Heterogeneity: $Tau^2 = 0.11$; $Chi^2 = 6.37$, df = 5 (P = 0.27); $I^2 = 21\%$

Test for overall effect: Z = 0.28 (P = 0.78)

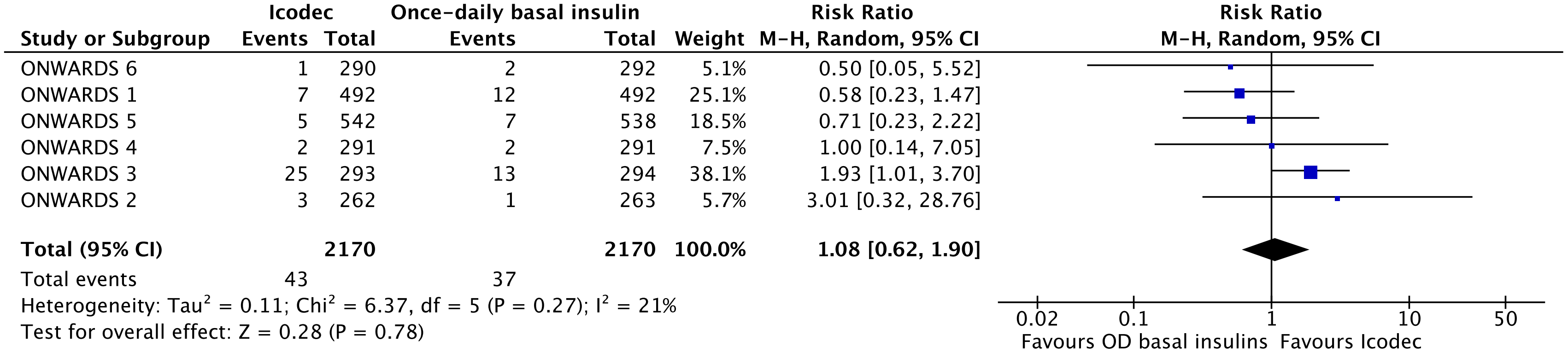

Abbreviations: ERR, Estimated Risk Ratio; OD, Once-Daily.
